# Application of the full-width-at-half-maximum image segmentation method to analyse retinal vascular changes in patients with internal carotid artery stenosis

**DOI:** 10.3389/fcell.2024.1467374

**Published:** 2024-08-19

**Authors:** Ligang Jiang, Mengting Liu, Meiting Yu, Wei Lu, Zhe Zhang, Yuhua Tong

**Affiliations:** ^1^ Quzhou Aliated Hospital of Wenzhou Medical University, Quzhou People’s Hospital, Quzhou, Zhejiang, China; ^2^ The Second Xiang Ya Hospital, Central South University, Changsha, Hunan, China; ^3^ Shenzhen Eye Institute, Shenzhen Eye Hospital, Jinan University, Shenzhen, China

**Keywords:** spectral domain optical coherence tomography (SD-OCT), full width at half Maximum, intelligent image analysis, internal carotid artery Stenosis, retinal arteriolar diameter, retinal venular diameter

## Abstract

**Background:**

To investigate the correlation between retinal vascular changes and ICA stenosis by measuring retinal vessels using full-width-at-half-maximum (FWHM) and intelligent image recognition.

**Methods:**

This research selected patients who were admitted to the Vascular Surgery Department of Quzhou People’s Hospital from January 2018 to December 2020 and were preparing for Carotid Artery Stenting (CAS). Participants were divided into two groups: without ICA stenosis (Group 0) and with ICA stenosis (Group 1). A total of 109 cases were included in the study, with 50 cases in Group 1 and 59 cases in Group 0. Vascular images of superior temporal zone B of the retina were obtained by spectral domain optical coherence tomography (SD-OCT). The edges of retinal vessels were identified by FWHM. Each vessel of all subjects was measured three times with the FWHM, and the average value was taken to obtain the retinal arteriolar lumen diameter (RALD), retinal arteriolar outer diameter (RAOD), retinal venular lumen diameter (RVLD), and retinal venular outer diameter (RVOD),Arterial Wall Thickness (AWT),Venular Wall Thickness (VWT)=(RVOD-RVLD)/2,Arteriovenous Ratio (AVR) = RAOD/RVOD.

**Results:**

We found that compared to Group 0, Group 1 had smaller RALD (*P* < 0.001) and RAOD (*P* < 0.001), and wider RVOD (*P* < 0.001), with thicker VWT (*P* < 0.001). When compared with the contralateral eye in Group 1, the ipsilateral eye exhibited even smaller RALD,RAOD and AVR (*P* < 0.001, *P* < 0.001, *P* < 0.001). After CAS, the RALD,RAOD and AVR in Group 1 increased (*P* < 0.001, *P* < 0.001, *P* < 0.001),while the RVLD and RVOD decreased (*P* < 0.05, *P* < 0.001). Our research reveals a significant correlation between retinal vascular changes and internal ICA stenosis.

**Conclusion:**

Utilizing SD-OCT in conjunction with the FWHM,we achieved a non-invasive, intelligent, stable, and precise acquisition of data pertaining to retinal vessels. These findings underscore a significant correlation between alterations in retinal vascular structure and the presence of ICA stenosis, as demonstrated by our research.

## 1 Introduction

Generally, Internal Carotid Artery (ICA) stenosis or obstruction which can lead to severe ischemic cerebrovascular diseases, is also a significant cause of ischemic ocular diseases, affecting not only patients’ vision but also potentially leading to physical disability and sudden death. The incidence of ocular symptoms increases correspondingly when the degree of ICA stenosis exceeds 50%. Recent epidemiological surveys have revealed a concerning trend that the prevalence of carotid artery stenosis in the population aged 30 to 79 was about 1.5% in 2020, which shows a sharp increase of 59.13% compared to what in 2000, and about 5%–10% of people over the age of 70 suffer from carotid artery stenosis (Lee et al., 2019a). This rate significantly rises with age and is higher in males than in females, posing a serious challenge to global public health.

Supplied by the Ophthalmic Artery (OA), the Central Retinal Artery (CRA) is a major intracranial branch of the ICA. The retinal blood vessels share similarities with cerebral blood vessels in embryonic origin, anatomical structure, and pathophysiology. Therefore, the changes of retinal blood vessels provide important clues for the diagnosis of cardiovascular and cerebrovascular diseases. Considering that the retinal blood vessels are the only blood vessel system in the human body that can be directly observed, they can not only effectively evaluate the functional state of the systemic microvasculature but also provide a direct and convenient approach of observation for clinical research on atherosclerosis in the head and neck arteries (London et al., 2013; Cheung et al., 2015).

Carotid atherosclerotic disease has become a significant burden on the global economy and healthcare systems, especially ICA stenosis. Therefore, timely and effective early intervention is crucial for reducing the incidence of ICA stenosis. For measuring the luminal diameter of the retinal blood vessels, this study adopts advanced technology called Spectral Domain Optical Coherence Tomography (SD-OCT), combined with Full-Width-at-Half-Maximum (FWHM) for intelligent image recognition and quantitative analysis. The purpose of the study is to explore the correlation between changes in retinal blood vessels and ICA stenosis, and provide an innovative, accurate, objective, and quantitative monitoring method for early diagnosis, risk assessment, continuous monitoring, and therapeutic decision-making of diseases. This method is expected to become a vital tool for improving the management and prevention strategies of ICA stenosis, providing the scientific basis for reducing the occurrence of related cardiovascular and cerebrovascular diseases.

## 2 Methods

### 2.1 General data and grouping

The subjects of this study were patients who were admitted to the Vascular Surgery Department of Quzhou People’s Hospital from January 2018 to December 2020, preparing for Carotid Artery Stenting (CAS). The study was approved by the Research Ethics Committee of Quzhou People’s Hospital and was conducted in accordance with the tenets of the Declaration of Helsinki, with informed consent obtained from all the participants. Enrolment criteria for the experimental group were as follows:1. Confirmed diagnosis of unilateral ICA stenosis (stenosis rate >50%), and no significant stenosis on the contralateral ICA by color doppler ultrasound of the head and neck and Computed Tomographic Angiography (CTA) to determine the degree of ICA stenosis. 2. Clear and visible fundus vessels. 3. No medication affecting vascular diameter such as angiotensin inhibitors or vasodilators had been taken within a month. 4. The best corrected visual acuity was ≥0.1: intraocular pressure <22 mmHg, refractive error < −3.00 diopters, no pathological high myopia. 5. No uveitis, glaucoma, retinal diseases, or any other diseases affecting retinal vessels and neural tissues. 6. No history of retinal photocoagulation: no history of intraocular surgery; 7. No macular edema or macular degeneration; 8. The vertical and horizontal cup-to-disk ratios of the optic disc were both <0.6, with a bilateral asymmetry rate <0.2; no pathological optic disc abnormalities; 9. No neurodegenerative diseases, such as Alzheimer’s disease, Parkinson’s disease, or dementia; 10. No pathological optic disc abnormalities. Exclusion criteria were as follows: 1. Severe liver and kidney dysfunction, thyroid diseases; 2. Various acute and chronic infectious diseases combined with autoimmune diseases, connective tissue diseases, and malignant tumors; 3. Severe myocardial diseases or valvular heart diseases; 4. Those with cerebrovascular accidents or other neurological diseases, as well as those with recent trauma, surgery, or pregnancy. Participants were divided into two groups: without ICA stenosis (Group 0), ICA stenosis (Group 1). A total of 109 patients were included in the study with 50 cases in Group 1 and 59 cases in Group 0. Group 1 performed ocular SD-OCT examination 1 day before CAS and 1 month after respectively.

### 2.2 Experimental procedures

#### 2.2.1 Acquisition of OCT images of retinal blood vessels

With the informed consent of the patients, examinations were conducted by using a Heidelberg SD-OCT instrument (Heidelberg Engineering, Inc., Heidelberg, Germany). The scanning light source wavelength was 870 nm; the fundus confocal laser imaging wavelength was 820 nm; the scanning speed was 40,000 A-Scans per second; the axial resolution was 5 μm; the transverse resolution was 6 μm; and the scanning depth was 2.0 mm. All examinations and measurements were performed by the same person. The ICA stenosis group performed ocular SD-OCT examinations 1 day before the CAS and 1 month after the surgery. The normal control group data was obtained from individuals who conducted normal physical examination, with consent from those individuals and their family members.

**FIGURE 1:**
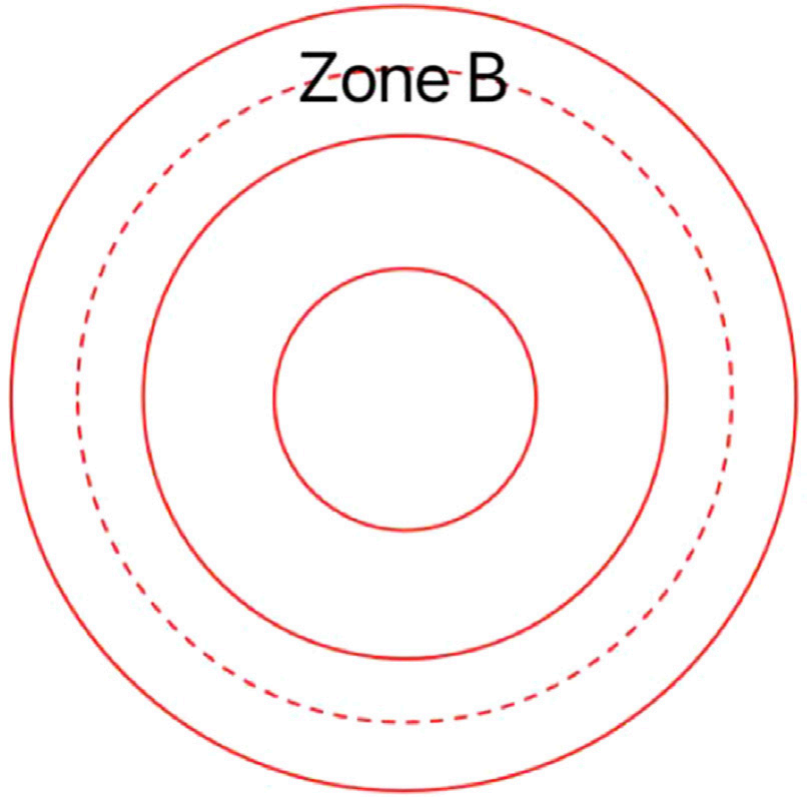
This is a transparent custom positioning ring used for locating zone B.

**FIGURE 2:**
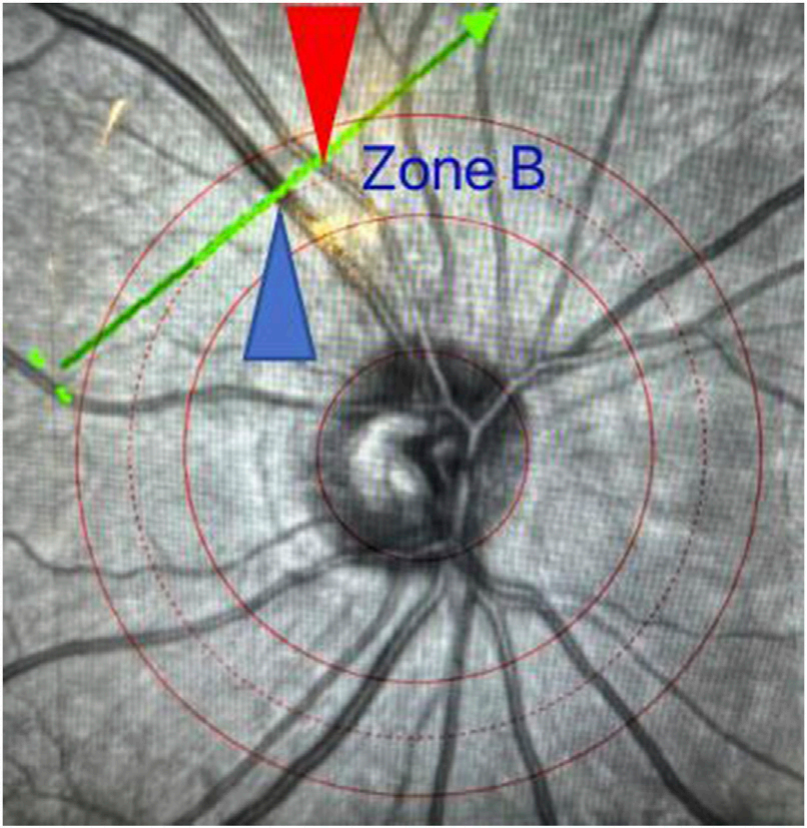
Schematic diagram of zone B positioning. The scanning line runs perpendicular to the axis of a retinal vessel in zone B with the red arrow indicating the artery and the blue arrow indicating the vein.

The specific operation is as follows: The patient sits in front of the SD-OCT device, places their head on the chin rest, and focuses on the cursor in the scanner. A specially designed positioning ring is used to locate the fundus retinal vascular scanning (Figure 1). The scanning mode is set to volume scan, with the parameter set to 284 A-scans. The scanning area is first determined to be the zone B (Figure 2). According to morphology, the characteristics of the blood vessels in zone B were more consistent with the description of arterioles and venules. Furthemore, after the retinal vessels emanate from the center of optic disc, there are fewer arteriovenous crossings and retinal arterial pulsation phenomena in the zone B, which do not affect the measurement of the diameters of the retinal artery or veins. First, create concentric circles on a transparent plastic film to determine the zone B. Based on the magnification of the fundus image from the SD-OCT, the central circle was used to determine the diameter of the optic disc, and then two concentric circles were drawn with diameters of 1 optic disc and 1.5 optic discs. The second and third circle diameters were the zone B. Scan the blood vessels in the zone B. The plastic film with the drawn positioning ring was attached to the computer screen, reseacher adjusted its position to be centered on the optic disc, and determined the location of the zone B. Scan the blood vessels in this area. If the vessel had branched before reaching zone B, then the part before branching was scanned. After scanning, the vertical-to-horizontal ratio of the obtained OCT images was first adjusted to 1:1 μm, the image was magnified to 800%, and then the OCT image was saved in BMP format. Only the images that clearly showed the vessel wall were used for the following analysis. At least five clearly scanned OCT images of every vessel were taken for analysis.

#### 2.2.2 Methods of measuring retinal vessels

FWHM is an image segmentation technique used for edge recognition in images. Due to the significant changes in grayscale at the edges of images, this algorithm can effectively identify these edges, thereby achieving image segmentation. Therefore, when applied to the identification of retinal vessel edges in OCT images, this method is less influenced by the image signals of surrounding tissues with high accuracy. Under ideal conditions, the inner and outer edges of the vessels are clear, but SD-OCT imaging can blur the edges, and the position of the vessel edges cannot be determined from a single image. Their positions are between the maximum and minimum grayscales passing through the edges, thus, we can obtain related data such as the inner and outer diameters of the retinal vessels by calculation.

**FIGURE 3:**
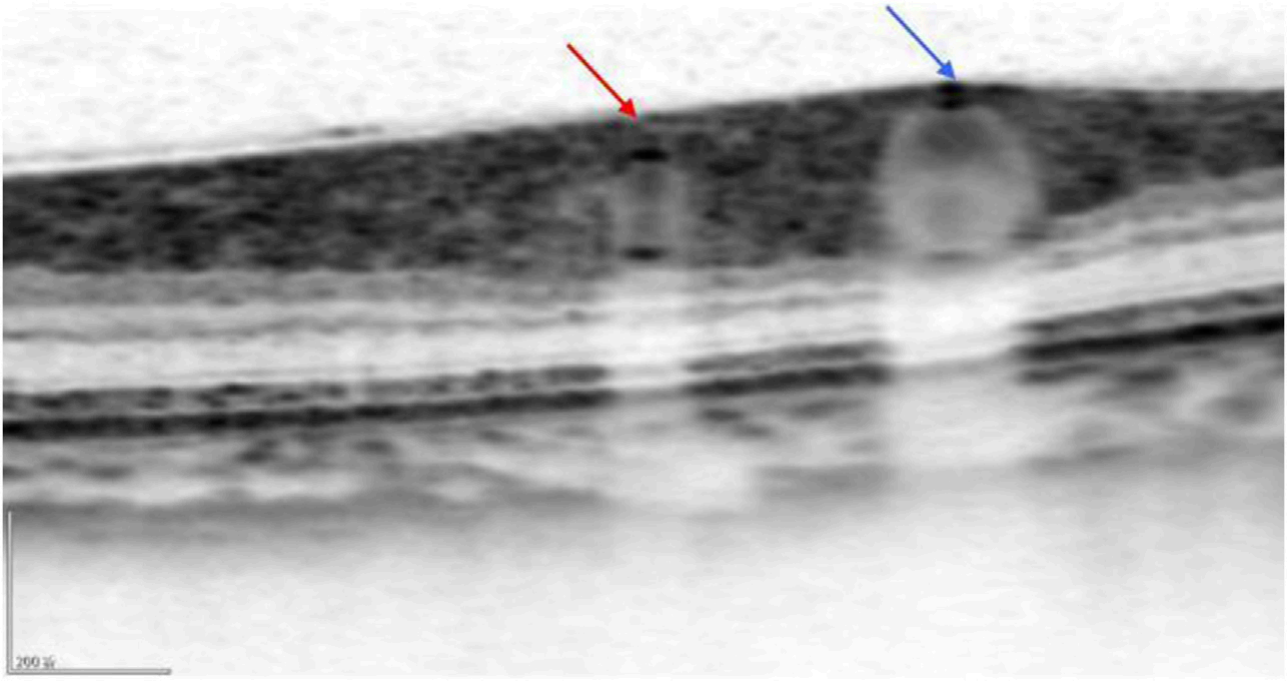
The OCT image clearly shows the cross-sections of the retinal artery (red arrow) and the retinal vein (blue arrow).

**FIGURE 4:**
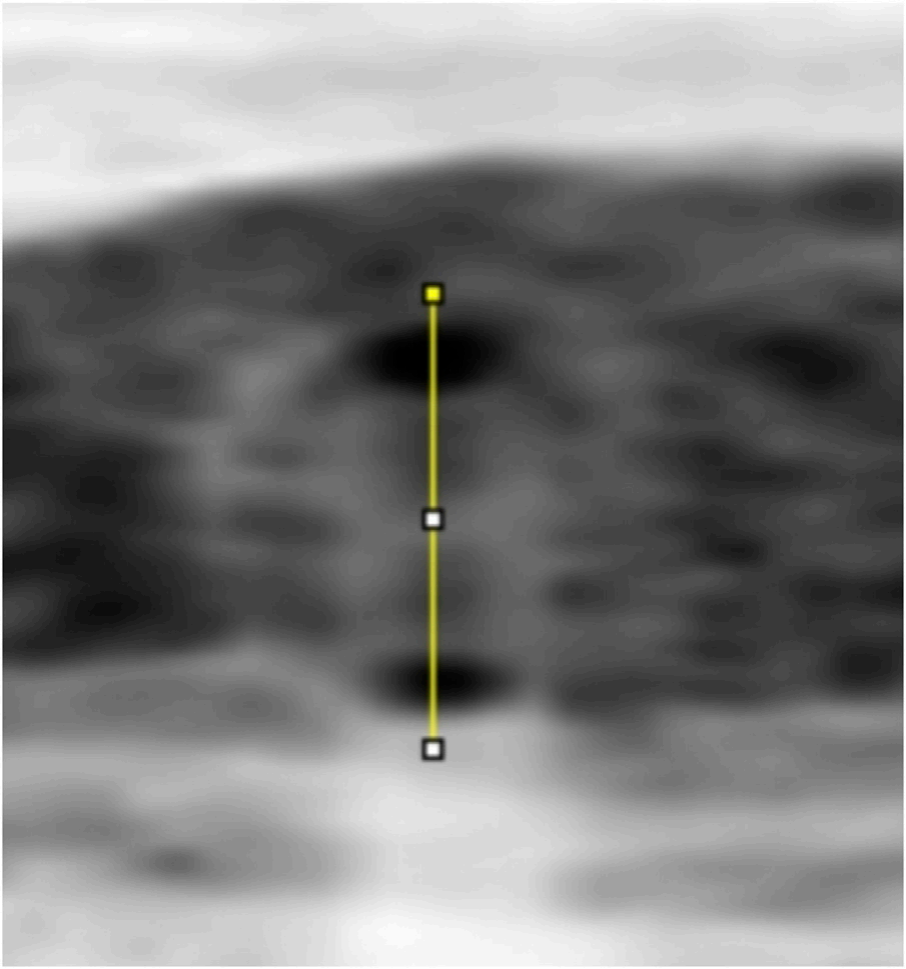
A line through the centre of the circle drawn between the upper and lower walls of a vessel to produce a greyscale density curve of the vessel wall.

**FIGURE 5:**
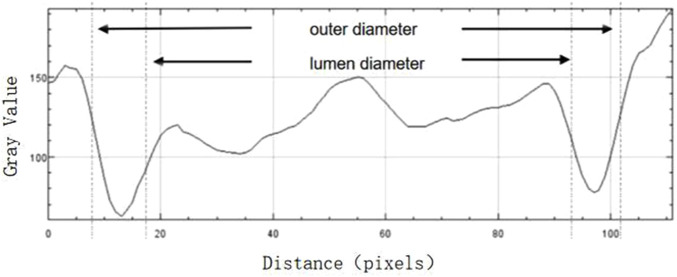
The boundaries of the upper and lower walls of the vessel determined with the FWHM segmentation method (shown by arrows), and the luminal and outer diameters of the vessel are calculated after the boundaries are determined.

FWHM identifies the edges of the vessel walls in OCT cross-sectional images, as shown in Figure 3. The OCT images in BMP format were opened in the ImageJ software (National Institutes of Health), and the line tool was used to draw a vertical line through the middle of the blood vessel to obtain the density curve (Figure 4). There are two upward-opening curves on the density curve, representing the upper and lower walls of the vessel in the OCT image. On both sides of each curve, he maximum and minimum values of the peaks and troughs were determined by the average of three consecutive values, and the median value between the maximum and minimum values was calculated (Figure 5). On each side of the curve, a linear function was fitted with the largest difference between continuous points, and the intersection of this linear function with the horizontal line of the median value was the position of the edge. Finally, the ImageJ software will automatically identify the distance between the boundary points of the two curves and calculate the inner and outer diameters of the retinal vessels, and obtain more parameters related to retinal vessels. The specific vascular structural parameters were as follows: (1) Retinal Arteriolar Lumen Diameter (RALD); (2) Retinal Arteriolar Outer Diameter (RAOD); (3) Arterial Wall Thickness (AWT) = (RAOD - RALD)/2; (4) Retinal Venular Lumen Diameter (RVLD); (5) Retinal Venular Outer Diameter (RVOD); (6) Venular Wall Thickness (VWT) = (RVOD - RVLD)/2; (7) Arteriovenous Ratio (AVR) = RAOD/RVOD. All procedures were performed by the same experienced ophthalmologist. The retinal vessels in the superotemporal zone of patients were selected for image collection, because of the thicker retinal vessels in the superotemporal zone is more consistent with the anatomical characteristics of small vessels with unconspicuous branches. Moreover, those vessels are further away from the optic disc on the nasal side, which can be less affected by vascular pulsation during measurement. Each vessel of all patients was measured three times through the above method, and the average value was taken. Previous research has also confirmed that FWHM could significantly reduce the deviation of repeated measurements compared to manual measurement, which improves the accuracy of vessel measurement (Tong et al., 2015).

#### 2.2.3 Statistical analytical methods

SPSS 25.0 was used for conducting statistical analysis. Quantitative data were subjected to the Shapiro-Wilk test for normality. Data that conform to a normal distribution were described by using the mean ± standard deviation, and intergroup comparisons were conducted by the independent samples t-test. Paired t-tests were used for related data from the same research subjects. Skewed data were described by the median (interquartile range), and intergroup comparisons used the rank sum test, and related data of the same research subjects took the paired rank sum test. Qualitative data were described as the number of cases (%), and intergroup comparisons conducted chi-square test or Fisher’s exact probability method. All statistical analyses were conducted by two-tailed tests with an alpha level of 0.05. *P* values < 0.05 were considered statistically significant.

## 3 Results

### 3.1 Comparison of general data

Group 1 (50 participants) and Group 0 (59 participants) showed no significant differences in terms of age, gender, diabetes, smoking, hypertension, dyslipidemia, and Body Mass Index (BMI) (*P* > 0.05). (Table 1).

**TABLE 1:** General clinical characteristics of patients in the two groups.

		Group 0	Group 1	*T* value*/*χ^*2*^value	*P* value
Age		62.31 ± 8.11	65.66 ± 9.53	−1.986	0.050
Sex	Male	23 (62.16)	14 (37.84)	1.456	0.228
	Female	36 (50.00)	36 (50.00)		
Diabetes	Yes	23 (63.89)	13 (36.11)	2.062	0.151
	No	36 (49.32)	37 (50.68)		
Smoking	Yes	16 (50.00)	16 (50.00)	0.377	0.577
	No	43 (55.84)	34 (44.16)		
Hypertension	Yes	33 (47.14)	37 (52.86)	3.845	0.050
	No	26 (66.67)	13 (33.33)		
Hyslipidemia	Yes	14 (46.67)	16 (53.33)	0.925	0.335
	No	45 (56.96)	34 (43.04)		
BMI		23.31 ± 3.69	23.81 ± 2.9	−0.764	0.446

### 3.2 Comparison of retinal vascular parameters between the left and right eyes in group 0

In Group 0, the differences in retinal vascular parameters between the left and right eyes were assessed using a paired t-test (Table 2). The results of the test showed that there were no significant differences in RALD, RAOD, AWT, RVLD, RVOD, VWT, and AVR (*P* > 0.05).

**TABLE 2:** Comparison of retinal vascular parameters between the left and right eyes in Group 0.

	The left eye	The right eye	*T* value	*P* value
RALD	114.46 ± 9.79	114.67 ± 11.86	0.299	0.766
RAOD	147.33 ± 10.81	146.91 ± 12.36	−0.490	0.626
AWT	16.44 ± 2.21	16.12 ± 2.63	−1.151	0.254
RVLD	150.21 ± 16.52	149.91 ± 17.33	−0.302	0.764
RVOD	178.01 ± 16.6	177.49 ± 18.35	−0.505	0.615
VWT	13.9 ± 3.37	13.79 ± 3.16	−0.337	0.737
AVR	0.83 ± 0.07	0.83 ± 0.07	0.065	0.949

### 3.3 Comparison of the retinal vascular parameters in the ipsilateral eyes between group 0 and group 1 before CAS

The differences in retinal vascular parameters between the right eyes of Group 0 and the ipsilateral eyes of Group 1 before CAS were evaluated using independent samples t-tests (Table 3, 4). The results of the tests indicated that there were no significant differences in AWT and RVLD (*P* > 0.05). However, significant differences were observed in RALD, RAOD, RVOD, VWT and AVR (*P* < 0.05).

**TABLE 3:** Comparison of the retinal vascular parameters in the ipsilateral eyes between Group 0 and Group 1 before CAS.

	Group 1 ipsilateral eyes before CAS	Group 0 right eyes	*T* value	*P* value
RALD	103.96 ± 11.56	114.67 ± 11.86	4.752	<0.001
RAOD	135.42 ± 12.17	146.91 ± 12.36	4.870	<0.001
AWT	15.73 ± 2.46	16.12 ± 2.63	0.792	0.430
RVLD	155.25 ± 16.51	149.91 ± 17.33	−1.639	0.104
RVOD	186.84 ± 18.65	177.49 ± 18.35	−2.630	0.010
VWT	15.79 ± 3.65	13.79 ± 3.16	−3.071	0.003
AVR	0.73 ± 0.09	0.83 ± 0.07	6.440	<0.001

**TABLE 4:** Comparison of the retinal vascular parameters in the ipsilateral eyes between Group 0 and Group 1 before CAS.

	Group 1 ipsilateral eyes before CAS	Group 0 left eyes	*T* value	*P* value
RALD	103.96 ± 11.56	114.46 ± 9.79	5.131	<0.001
RAOD	135.42 ± 12.17	147.33 ± 10.81	5.409	<0.001
AWT	15.73 ± 2.46	16.44 ± 2.21	1.576	0.118
RVLD	155.25 ± 16.51	150.21 ± 16.52	−1.589	0.115
RVOD	186.84 ± 18.65	178.01 ± 16.6	−2.615	0.010
VWT	15.79 ± 3.65	13.9 ± 3.37	−2.815	0.006
AVR	0.73 ± 0.09	0.83 ± 0.07	6.496	<0.001

The differences in retinal vascular parameters between the left eyes of Group 0 and the ipsilateral eyes of Group 1 before CAS were evaluated using independent samples t-tests. The results showed no significant differences in AWT and RVLD (*P* > 0.05), while there were significant differences in RALD, RAOD, RVOD, VWT, and AVR (*P* < 0.05).

### 3.4 Comparison of the retinal vascular parameters in the ipsilateral eyes between group 0 and group 1 after CAS

In the comparison of retinal vascular parameters between the right eyes of Group 0 and the eyes of Group 1 after CAS, independent samples t-tests were used to evaluate the differences (Table 5, 6). The results of the tests showed that there were no significant differences in AWT, RVLD, and RVOD (*P* > 0.05). However, significant differences were found in RALD, RAOD, VWT, and AVR (*P* < 0.05).

**TABLE 5:** Comparison of retinal vascular parameters between ipsilateral and contralateral eyes in Group 1 after CAS.

	Group 1Ipsilateral eyes after CAS	Group 0 right eyes	*T* value	*P* value
RALD	108.02 ± 11.48	114.67 ± 11.86	2.958	0.004
RAOD	139.56 ± 12.26	146.91 ± 12.36	3.105	0.002
AWT	15.77 ± 2.38	16.12 ± 2.63	0.727	0.469
RVLD	150.82 ± 15.68	149.91 ± 17.33	−0.287	0.775
RVOD	182.23 ± 17.87	177.49 ± 18.35	−1.363	0.176
VWT	15.71 ± 4.12	13.79 ± 3.16	−2.740	0.007
AVR	0.77 ± 0.09	0.83 ± 0.07	3.759	<0.001

**TABLE 6:** Comparison of retinal vascular parameters between ipsilateral and contralateral eyes in Group 1 after CAS.

	Group 1Ipsilateral eyes after CAS	Group 0 left eyes	*T* value	*P* value
RALD	108.02 ± 11.48	114.46 ± 9.79	3.156	0.002
RAOD	139.56 ± 12.26	147.33 ± 10.81	3.515	0.001
AWT	15.77 ± 2.38	16.44 ± 2.21	1.519	0.132
RVLD	150.82 ± 15.68	150.21 ± 16.52	−0.198	0.843
RVOD	182.23 ± 17.87	178.01 ± 16.6	−1.279	0.204
VWT	15.71 ± 4.12	13.9 ± 3.37	−2.515	0.013
AVR	0.77 ± 0.09	0.83 ± 0.07	3.698	<0.001

In the comparison of retinal vascular parameters between the left eyes of Group 0 and the ipsilateral eyes of Group 1 after CAS, independent samples t-tests were utilized to evaluate the differences. The findings indicated that there were no significant differences in AWT, RVLD, and RVOD (*P* > 0.05). In contrast, significant differences were observed in RALD, RAOD, VWT, and AVR (*P* < 0.05).

### 3.5 Comparison of retinal vascular parameters between ipsilateral and contralateral eyes in group 1 before and after CAS

In the comparison of retinal vascular parameters between the ipsilateral and contralateral eyes of Group 1 before CAS, paired sample t-tests were used to assess the differences (Table 7, 8). The results indicated that there were no significant differences in AWT, RVLD, RVOD, and VWT (*P* > 0.05). However, significant differences were noted in RALD, RAOD, and AVR (*P* < 0.05).

**TABLE 7:** Comparison of retinal vascular parameters between ipsilateral and contralateral eyes in Group 1 before CAS.

	Group 1 ipsilateral eyes before CAS	Group 1 contralateral eyes before CAS	*T* value	*P* value
RALD	103.96 ± 11.56	111.18 ± 10.23	−9.361	<0.001
RAOD	135.42 ± 12.17	143.4 ± 9.03	−6.862	<0.001
AWT	15.73 ± 2.46	16.11 ± 2.6	−0.830	0.411
RVLD	155.25 ± 16.51	152.67 ± 17.92	0.956	0.344
RVOD	186.84 ± 18.65	183.45 ± 18.41	1.132	0.263
VWT	15.79 ± 3.65	15.39 ± 3.14	0.645	0.522
AVR	0.73 ± 0.09	0.79 ± 0.09	−4.247	<0.001

**TABLE 8:** Comparison of retinal vascular parameters between ipsilateral and contralateral eyes in Group 1 after CAS.

	Group 1 ipsilateral eyes after CAS	Group 1 contralateral eyes after CAS	*T* value	*P* value
RALD	108.02 ± 11.48	110.47 ± 10.69	−3.045	0.004
RAOD	139.56 ± 12.26	143.6 ± 9.09	−3.449	0.001
AWT	15.77 ± 2.38	16.56 ± 2.82	−1.713	0.093
RVLD	150.82 ± 15.68	147.34 ± 18.65	1.157	0.253
RVOD	182.23 ± 17.87	179.48 ± 18.17	0.859	0.394
VWT	15.71 ± 4.12	16.07 ± 4.00	−0.548	0.586
AVR	0.77 ± 0.09	0.81 ± 0.09	−2.150	0.036

In the comparison of retinal vascular parameters between the ipsilateral and contralateral eyes of Group 1 after CAS, paired sample t-tests were used to evaluate the differences. The results indicated that no significant differences in AWT, RVLD, RVOD, and VWT) (*P* > 0.05). In contrast, significant differences were identified in RALD, RAOD, and AVR (*P* < 0.05).

### 3.6 Comparison of retinal vascular parameters in ipsilateral eyes before and after CAS in group 1

In the comparison of retinal vascular parameters in the ipsilateral eyes of Group 1 before and after CAS, paired sample t-tests were utilized to determine the significance of differences (Table 9). The findings indicated that there were no significant changes in AWT and VWT (*P* > 0.05). In contrast, significant differences were noted in RALD, RAOD, RVLD, RVOD, and AVR (*P* < 0.05).

**TABLE 9:** Comparison of retinal vascular parameters in ipsilateral eyes before and after CAS in Group 1.

	Group 1 ipsilateral eyes before CAS	Group 1 ipsilateral eyes after CAS	*T* value	*P* value
RALD	103.96 ± 11.56	108.02 ± 11.48	−14.785	<0.001
RAOD	135.42 ± 12.17	139.56 ± 12.26	−13.177	<0.001
AWT	15.73 ± 2.46	15.77 ± 2.38	−0.212	0.833
RVLD	155.25 ± 16.51	150.82 ± 15.68	3.712	0.001
RVOD	186.84 ± 18.65	182.23 ± 17.87	4.935	<0.001
VWT	15.79 ± 3.65	15.71 ± 4.12	0.202	0.841
AVR	0.73 ± 0.09	0.77 ± 0.09	−10.995	<0.001

## 4 Analysis and discussion

As is well known, ICA stenosis may cause severe ischemic cerebrovascular diseases, and the correlation between asymptomatic ICA stenosis and silent cerebral infarction might be related to the degree of stenosis. ICA stenosis can directly affect the blood supply to the eyes, leading to Ocular Ischemic Syndrome (OIS), which is caused by insufficient ocular perfusion due to ipsilateral common carotid artery stenosis or ICA stenosis. The mortality rate of OIS is as high as 40% within a few years after onset (Terelak-Borys et al., 2012), and approximately 66% of the main causes of death are cardiovascular diseases, followed by stroke. The most common symptom of OIS is vision loss, some of which are acute and irreversible. Retinal vascular changes are often asymptomatic and up to 29% of patients with carotid artery occlusion would suffer from these lesions, with 1.5% chance of progressing to symptomatic OIS annually (Mendrinos et al., 2010). When the degree of ICA stenosis is greater than 50%, the incidence of ocular symptoms increases (Lawrence and Oderich, 2002). Therefore, early recognition of minor changes in the retina is particularly important for patients with ICA stenosis.

The retinal vessels play a role in evaluating the function of the systemic microvasculature and can provide detailed vascular information for the study of atherosclerosis in the head and neck. Subtle changes in vascular morphology have a certain predictive role in the warning of head and neck vascular diseases. Initially, most of studies mainly discussed the link between the severity of ocular ischemic symptoms or postoperative prognosis and ICA stenosis. For example, Klijn et al. (2002) conducted a prospective observational analysis of 110 patients with ICA stenosis and found that 32 patients with ocular symptoms had venous stasis retinopathy, with 4 eventually developing into OIS. Kawaguchi et al. (2006) examined 38 patients with ICA stenosis requiring CAS (stenosis degree >80%) and found that CAS could effectively improve ocular blood circulation and improve chronic OIS caused by severe ICA stenosis. Similarly, a study (Ma et al., 2018),which included 64 eyes of 64 patients with OIS and ipsilateral ICA ≥70%, assessed the blood flow in the eyes after CAS, and found that the Peak Systolic Velocity (PSV) of the OA, CRA, and short posterior ciliary artery significantly increased at 1 month, 3 months, 6 months, and 12 months post-surgery, which indicated a clear improvement in blood flow after the operation. Subsequently, thanks to the rapid development of color doppler ultrasound imaging technology and probes, the application of Color Doppler Flow Imaging (CDFI) technology can clearly distinguish ocular vessels and evaluate their hemodynamic changes, and has become a reliable non-invasive method for examining carotid artery diseases and ocular ischemic diseases. Drakou et al. (2011) thoroughly discussed the changes in OA blood flow caused by severe ICA stenosis and the impact of carotid revascularization on the eyes and brain circulation. The results showed that as the degree of ICA stenosis increased, the PSV of the OA decreased, but in severe ICA, blood flow could not be detected, and there might be reverse flow. Heßler et al. (2015) performed head and neck ultrasound examinations on 27 patients with severe ICA stenosis and found that the PSV values of the CRA showed a significant downward trend with the aggravation of stenosis, and the degree of decline was significantly correlated with the degree of stenosis to some extent. Lee et al. (2019b) used Optical Coherence Tomography Angiography (OCTA) to assess the impact of CAS on retinal vessels in patients with severe ICA stenosis, and found that after CAS, the vascular density in the macular area of both eyes increased, which revealed that the blood supply gradually recovered after the relief of ICA stenosis. The above studies only discussed the correlation between retinal vessels and ICA stenosis in terms of the hemodynamics of the OA, CRA, and macular blood density, and there has been no relevant research and analysis about retinal vessel diameter. Moreover, the display of the skull base and intracranial vessels by ultrasound examination is limited, and the diagnostic accuracy is also affected by the operation technique and personal diagnostic experience. In addition, the structure of the retinal vessels may have undergone subtle changes before, and these assessment results are greatly influenced by human factors and cannot be accurately and quantitatively evaluated.

As the only blood vessels in human body that can be directly, repeatedly and non-invasively observed, retinal blood vessels have the similarities between cerebral vessels in embryonic origins, anatomical structures, and pathophysiological foundations. Retinal blood vessels can effectively assess the function of systemic microvasculature, and provide a convenient approach for the study of atherosclerosis in the head and neck. The most direct and objective indicator of observation for retinal blood vessels is the change in the caliber of retinal vessels. However, there are few methods for monitoring retinal vessels. Previous studies have reported that the morphology of retinal vessels can be tracked by various methods, including ophthalmoscopy, fluorescein angiography, and fundus photography, but the results are greatly influenced by human factors and cannot be precisely quantified. There still are demands for more accurate and objective quantitative detection techniques. In epidemiological surveys, many large institutions use fundus photography and employ semi-automatic vessel measurement software centered on the optic disc to obtain fundus images. By imaging the vascular area of 0.5D to 1.0D from the edge of the optic disc, six of the most prominent arterioles and venules are measured, thereby converting to data on the central retinal artery equivalent (CRAE) and central retinal vein equivalent (CRVE). In recent years, research on retinal vessels in the fundus has gradually evolved towards a more detailed and intelligent direction. Researchers have begun to explore the use of deep learning models to automatically measure retinal vessel parameters in fundus photographs to predict the occurrence of cardiovascular events (Cheung et al., 2020). Additionally, there is a research team that has adopted a system called Eye Art, which is a cloud-based automatic artificial intelligence eye screening technology (Bhaskaranand et al., 2019). It is capable of collecting and analyzing the retinal images of patients.

This study adopts an innovative measurement methods that combines SD-OCT with FWMH to achieve the quantitative analysis for the retinal blood vessel. SD-OCT is an essential imaging tool in retinal tomography analysis. It is renowned for its high precision, clear imaging quality, dynamic continuous tomographic analysis capabilities, and non-invasive nature. In addition, it has the ability to track eye movement, stably locking onto the target blood vessels even as the eye rotates. These features make SD-OCT an ideal tool for analyzing retinal vessels and obtaining vascular tomographic images, and provide a more intuitive and convenient method of analysis. By using SD-OCT to dissect retinal vessels, we can obtain high-definition vascular tomographic images, which can not only provide a basis for in-depth research on vascular, but also facilitate precise repositioning and detection of specific vessels. The application of this technology has significantly improved the accuracy and reliability of our diagnoses of retinal vascular diseases, and laid the solid foundation for further research and clinical application. FWHM is an edge-based segmentation method that fundamentally relies on changes in the grayscale values of edge pixels to segment and recognize images. It can determine blood vessel boundaries with lower sensitivity to noise points and interference from adjacent tissues more quickly and stably (Tong et al., 2015). Micro-density image intelligent segmentation technology is an important technique that divide images into several meaningful parts based on features such as texture, grayscale, and color in the medical image processing field. The properties of these parts might be similar or contrasting, with the true purpose being to extract the “regions of interest” from the image to provide relevant assistance for clinical diagnosis. Our team has made significant breakthroughs in the research fields of systemic diseases such as diabetes, hypertension, and coronary heart disease. We have successfully validated the feasibility and scientificity of using this technology to measure retinal vessels. By precisely measuring retinal vessels, we can further comprehend the correlation between these systemic diseases and retinal vessels. The innovation of this study is to achieve precise measurements of retinal lumen diameter and outer diameter on SD-OCT images by combining FWHM with micro-density image intelligent recognition. This technology has obtained a national invention patent and offers greater accuracy compared to traditional methods of measuring retinal central artery and vein equivalents.

A population-based cross-sectional study (Liao et al., 2004) involving 8,031 men and women aged between 45 and 64 years examined the association between carotid artery stiffness (a marker of early atherosclerosis) and the narrowing of retinal arterioles. It was found that an increase in carotid stiffness was associated with the narrowing of retinal arterioles, which may lead to the narrowing of retinal arterioles by increasing systolic blood pressure and thus increasing carotid stiffness. The link between carotid stiffness and the narrowing of systemic small arteries may provide important insights into the interrelationship between the processes of large and small vessel diseases involved in cerebrovascular diseases, and relatively directly indicates a certain correlation between carotid atherosclerosis and retinal arterioles. Another cross-sectional study (Wu et al., 2022), which involved 41 patients with varying degrees of ICA stenosis, used computer-assisted vascular analysis software to analyze CRAE, CRVE, and AVR. It showed that patients with ipsilateral ICA stenosis had smaller CRAE and AVR, which is consistent with our research findings. In our study, patients with ICA stenosis had smaller RALD and RAOD compared to the normal control group, and after CAS, the RALD and RAOD of patients with ipsilateral ICA stenosis widened, with improved blood flow in the neck.

Larger retinal venular diameters are associated with risk factors for cardiovascular diseases, independent of other cardiovascular risk factors, such as atherosclerosis, carotid artery stenosis, higher levels of total cholesterol, lower levels of high-density lipoprotein, higher levels of inflammatory markers, and smoking (Ikram et al., 2004a). These factors are also related to the risk of stroke. Even after adjusting for all these risk factors, the relationship between venous dilation and stroke still exists, which indicates that there are other mechanisms affecting the dilation of retinal veins. Some scholars (Klein et al., 2004) have discussed that retinal hypoxia may cause the increasing diameter of retinal venules, which is not only found in the early stages of diabetic retinopathy but also in venous stasis retinopathy, especially in carotid occlusive diseases. Insufficient perfusion of the brain and retina seems to be an important factor in the development of this type of venous stasis retinopathy. Carotid endarterectomy or retinal photocoagulation treatment may improve retinal hypoxia. Perhaps, the dilation of retinal venules is a general characteristic of reflecting reduced blood flow and diffuse cerebral ischemia. It has been reported (De Silva et al., 2009) that in a study of 1,029 patients with acute stroke, retinal venular diameter was related to ipsilateral ICA stenosis. Patients with wider retinal venular diameters were more likely to have severe ipsilateral carotid artery disease, which is unrelated to the contralateral side. Severe carotid artery disease can lead to ischemia and hypoxia of the retinal vascular wall, causing dilation of the retinal veins. Machalińska et al. (2019) found that after carotid endarterectomy, CRVE decreased in both eyes through assessing the CRVE in both eyes of 65 patients with unilateral ICA stenosis and 34 healthy subjects. In this study, RVLD and RVOD in the ICA stenosis group were wider, and after CAS, both of RVLD and RVOD decreased, which is basically consistent with the above views.

The ratio of the retinal artery diameter to the diameter of the equative accompanying retinal vein called AVR is a comprehensive indicator for evaluating retinal vessels. The ARIC study (Wong et al., 2001) suggests that this ratio could reflect information about cardiovascular diseases. Lower AVR is associated with an increased risk of stroke, and also correlated with a certain correlation with carotid atherosclerotic plaques and arteriosclerosis. However, this indicator has its limitations, as we cannot determine whether it is the retinal artery, the retinal vein, or the combined effect of both that influences the AVR. Some studies have indicated (Ikram et al., 2004b) that the diameter of retinal veins does not remain constant under different pathological conditions, so the retinal arteries and veins should be analyzed separately to reassess AVR. In this study, we concluded that the ICA stenosis group had a lower AVR. In the same subject with ICA stenosis, the AVR of the same-side eye before surgery was lower than that of the opposite eye, and the AVR of the same-side eye increased after surgery, which indicates relevance between ICA stenosis and AVR to some extent, which is similar to the conclusions of the aforementioned studies.

An additional breakthrough of this study lies in our acquisition of AWT and VWT measurements. Wong et al. (2002) conducted a comparative analysis of 106 hypertensive patients and 132 well-matched control subjects, finding that the average AWT and VWT in hypertensive patients were significantly higher than those in the age-matched normal population. AWT can be considered a more sensitive indicator for evaluating hypertensive retinopathy than retinal artery diameter. Currently, there are few research on the correlation between ICA stenosis and retinal vascular wall thickness. In this study, we attempted to explore the correlation between ICA stenosis and the thickness of retinal vascular walls. We found that in patients with ICA stenosis, VWT was relatively larger, which is consistent with some of the previous conclusions, while AWT showed no significant difference, which might be related to the small sample size. Further research will be conducted in the future.

There is relatively little research both domestically and internationally on ICA stenosis and its impact on changes in retinal vessel diameter. Common research methods are based on measuring the equivalents of the central retinal artery and vein. The advantage of our study lies in the use of the FWHM combined with SD-OCT technology for intelligent quantitative analysis of retinal vessel images. Moreover, our experimental subjects are all the same individual, and what we compare is the changes of retinal vascular structure of the same individual before and after CAS. This automatically excludes potential confounding factors such as age, gender, diabetes, smoking, hypertension, dyslipidemia, and BMI, thereby enhancing the accuracy and reliability of the research results.

This study has several limitations. First, compared to the other large-scale epidemiological research, the sample size of this study is relatively small. Second, the resolution of the OCT images obtained is relatively low, and we will use high-resolution OCTA equipment to collect OCT images in further studies. Third, patients with ICA stenosis may have collateral circulation, and it is currently difficult to determine the occurrence and duration of blood flow compensation, which may have a certain impact on the research results. Fourth, the dynamic changes in retinal vascular morphology require long-term follow-up to conduct in-depth analysis. Finally, while FWHM has effectively reduced measurement errors, it has not yet achieved full automation, and the efficiency of measurement needs to be improved. We have jointly developed a retinal vessel measurement software based on artificial intelligence in-depth learning, which is currently in the testing phase. This software is expected to significantly improve the accuracy of retinal vessel measurement, establish a large-scale database of retinal vessels in normal populations, and has important clinical significance and scientific value for evaluating ICA stenosis and atherosclerotic diseases of the head and neck vessels. More relevance between retinal vessels and systemic vascular diseases will also be further revealed and developed, providing strong support for the early prevention and treatment of systemic vascular diseases moreover through this software.

## 5 Conclusion

Utilizing SD-OCT and FWHM, we have successfully acquired pertinent data on retinal vessels in a non-invasive, intelligent, stable, and precise manner. Our findings indicate that patients with ipsilateral ICA stenosis exhibit reduced RALD, RAOD and AVR, After CAS, the RALD, RAOD and AVR widened, while the RVLD and RVOD decreased. In conclusion,the study demonstrates a significant correlation between retinal vascular alterations and ICA stenosis.

## Data Availability

The raw data supporting the conclusions of this article will be made available by the authors, without undue reservation.
